# Supplementary figures and images for: Anti-tau antibodies targeting a conformation-dependent epitope selectively bind seeds
Source: J Biol Chem. 2023 Sep 14;299(11):105252. doi: 10.1016/j.jbc.2023.105252 (PMC10582770; doi:10.1016/j.jbc.2023.105252)

**A**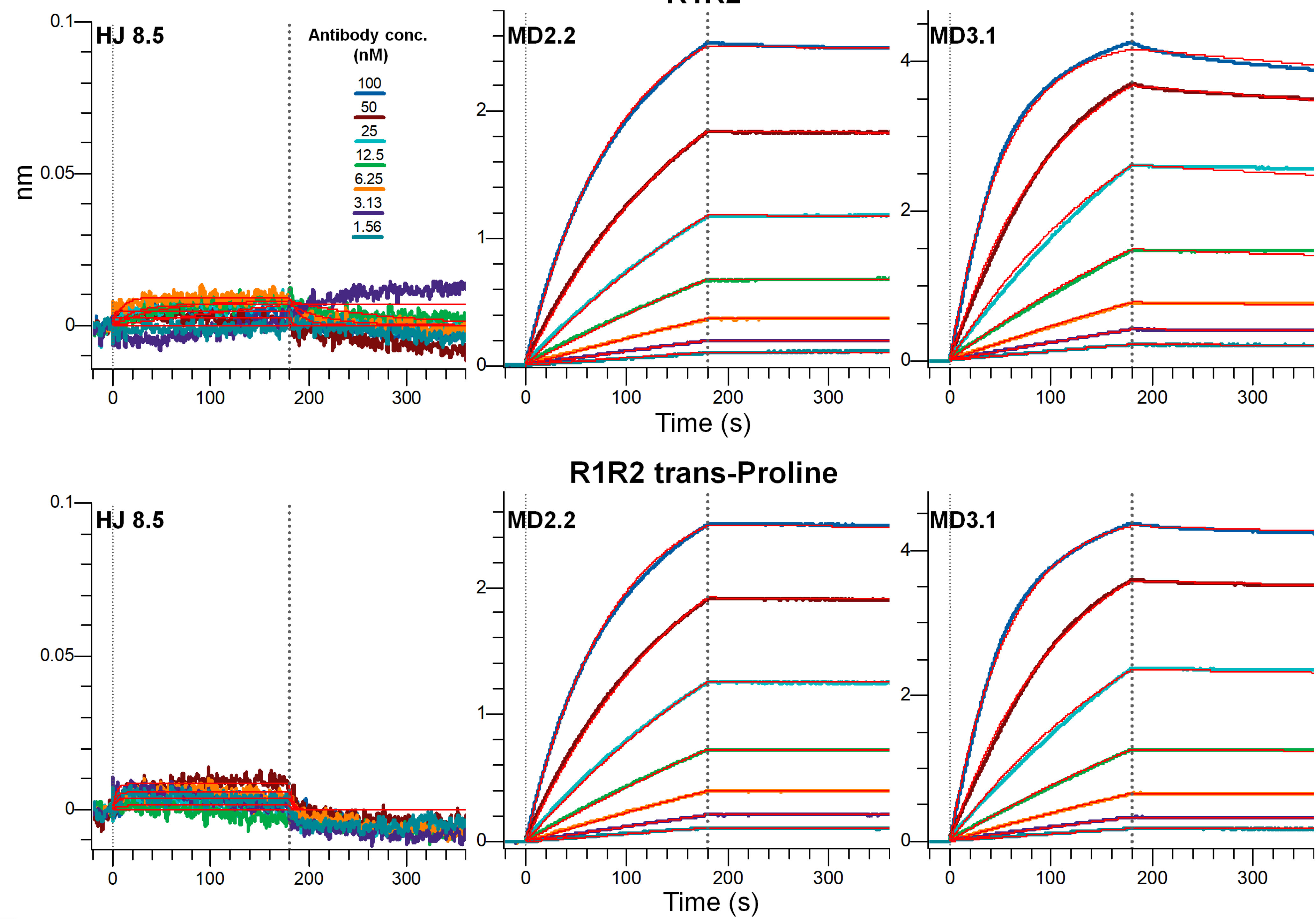**B**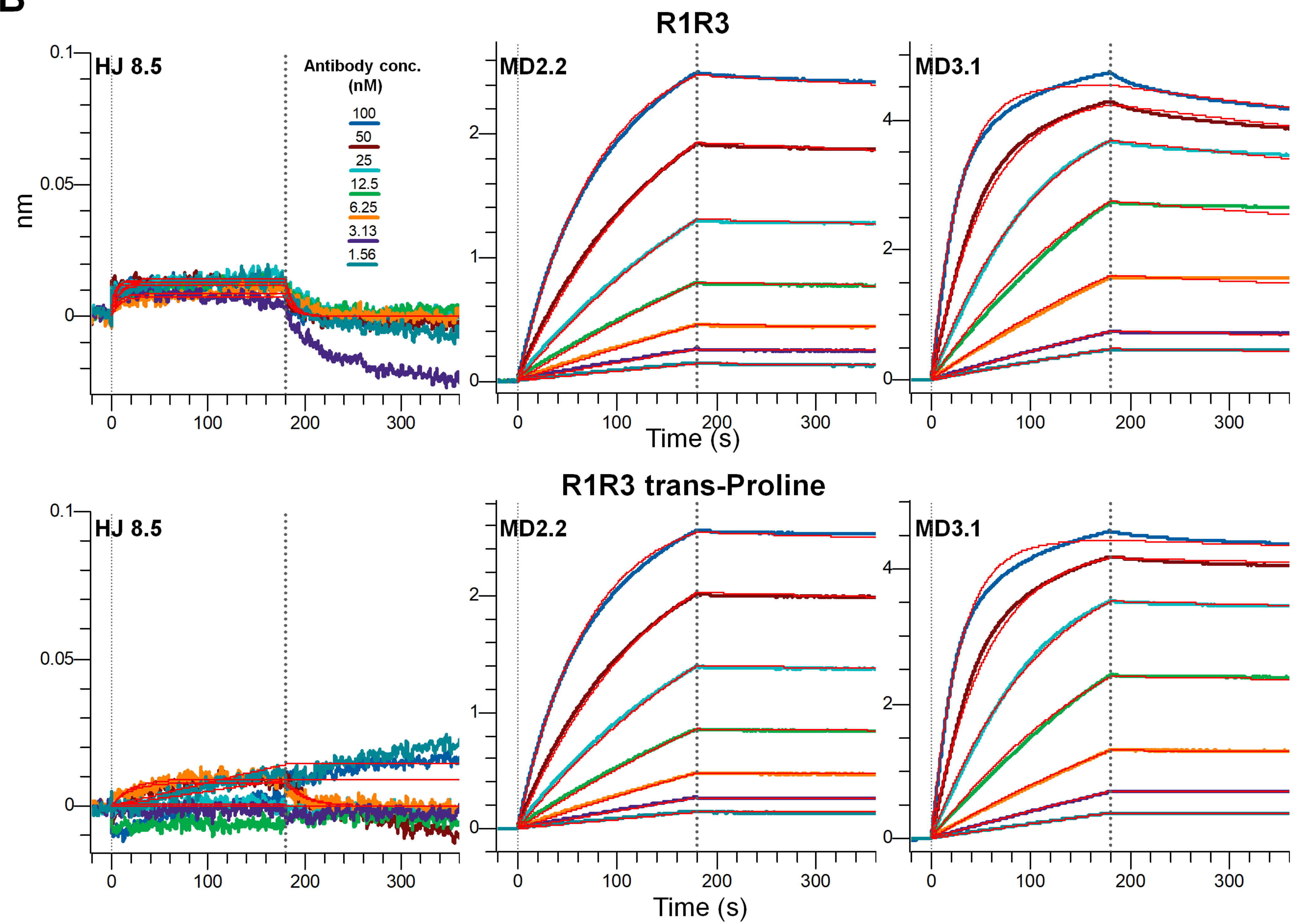

Supplement: Supporting Figure S1 — BLI kinetics curves of antibody binding to various R1R2/R1R3 tau peptides.A, kinetic traces for R1R2 (upper panel) and R1R2 trans-proline (bottom panel) peptides. The kinetic traces are fitted to 1:1 kinetics. B, (A) Kinetic traces for R1R3 (upper panel) and R1R3 trans-proline (bottom panel) peptides. The kinetic traces are fitted to 1:1 kinetics. Whereas both MD2.2 and MD3.1 bound peptides, we observed no significant binding with HJ8.5. [file mmc1.pdf]

**MD2.2**

**MD3.1**

**AD**

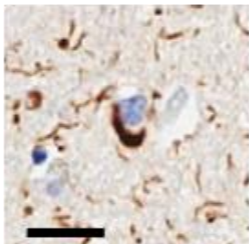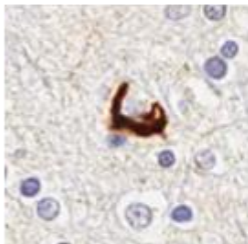

**PSP**

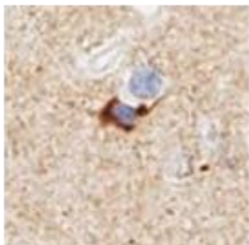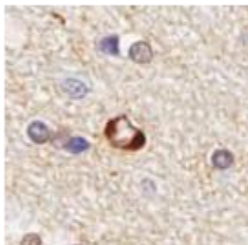

Supplement: Supporting Figure S2 — Expanded high-magnification images of MD2.2 and MD3.1-positive perinuclear inclusions in AD and PSP brain tissue. Scale bar = 20 μm. [file mmc2.pdf]

Figure 4A

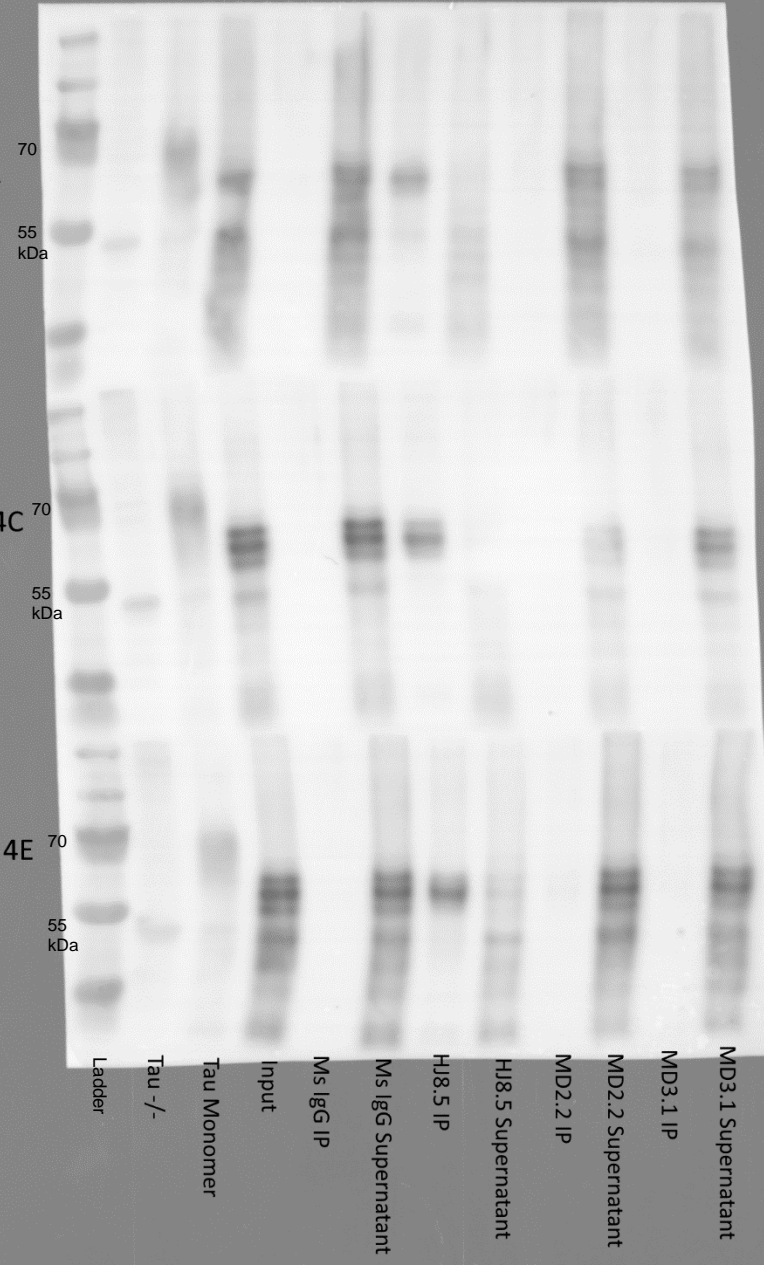

Figure 4C

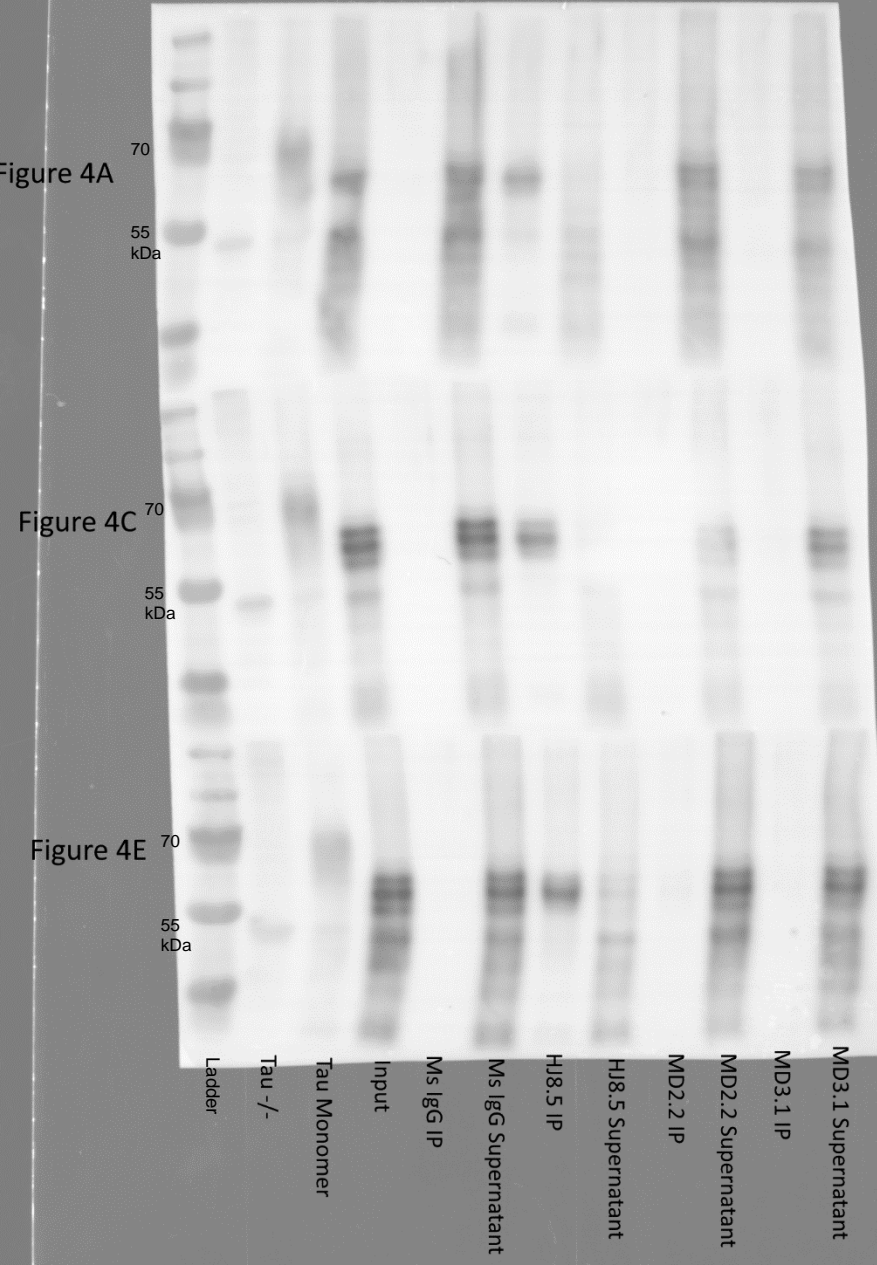

Figure 4E

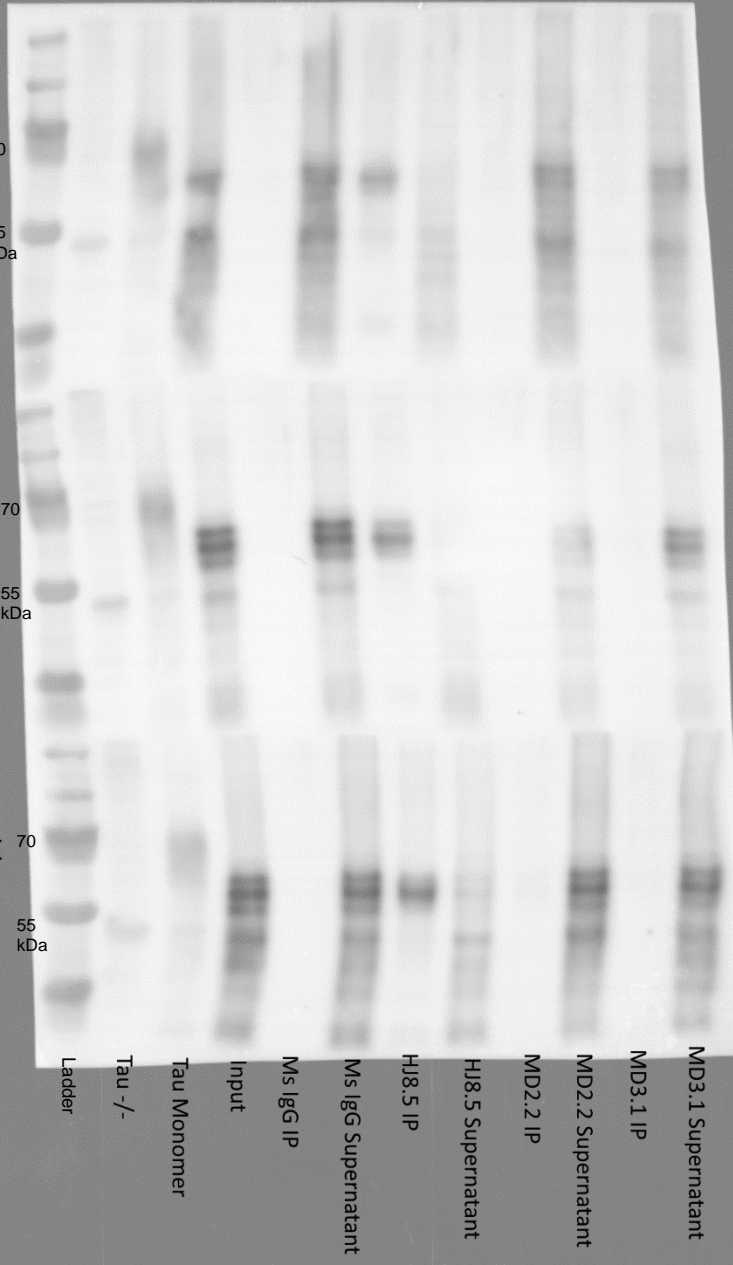

Supplement: Supporting Figure S4 — Unprocessed images of western blots used inFigure 4. [file mmc4.pdf]

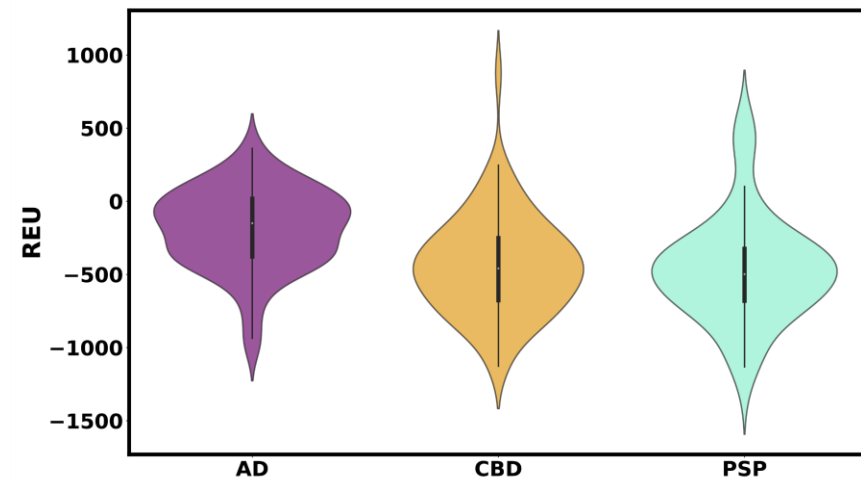

Supplement: Supporting Figure S5 — Energy distribution plot of assembled AD, CBD and PSP models in fibril form. Energy distribution of ensembles produced for AD, CBD and PSP trimers containing full length 2N4R tau. The energies are shown as Rosetta Energy Units (REU). [file mmc5.pdf]
