# Supplementary material for: Anti-tau antibodies targeting a conformation-dependent epitope selectively bind seeds
Source: J Biol Chem. 2023 Sep 14;299(11):105252. doi: 10.1016/j.jbc.2023.105252 (PMC10582770; doi:10.1016/j.jbc.2023.105252)
Supplement: Supporting Figure S3 — Unprocessed images of western blots used inFigure 3. [file mmc3.pdf]

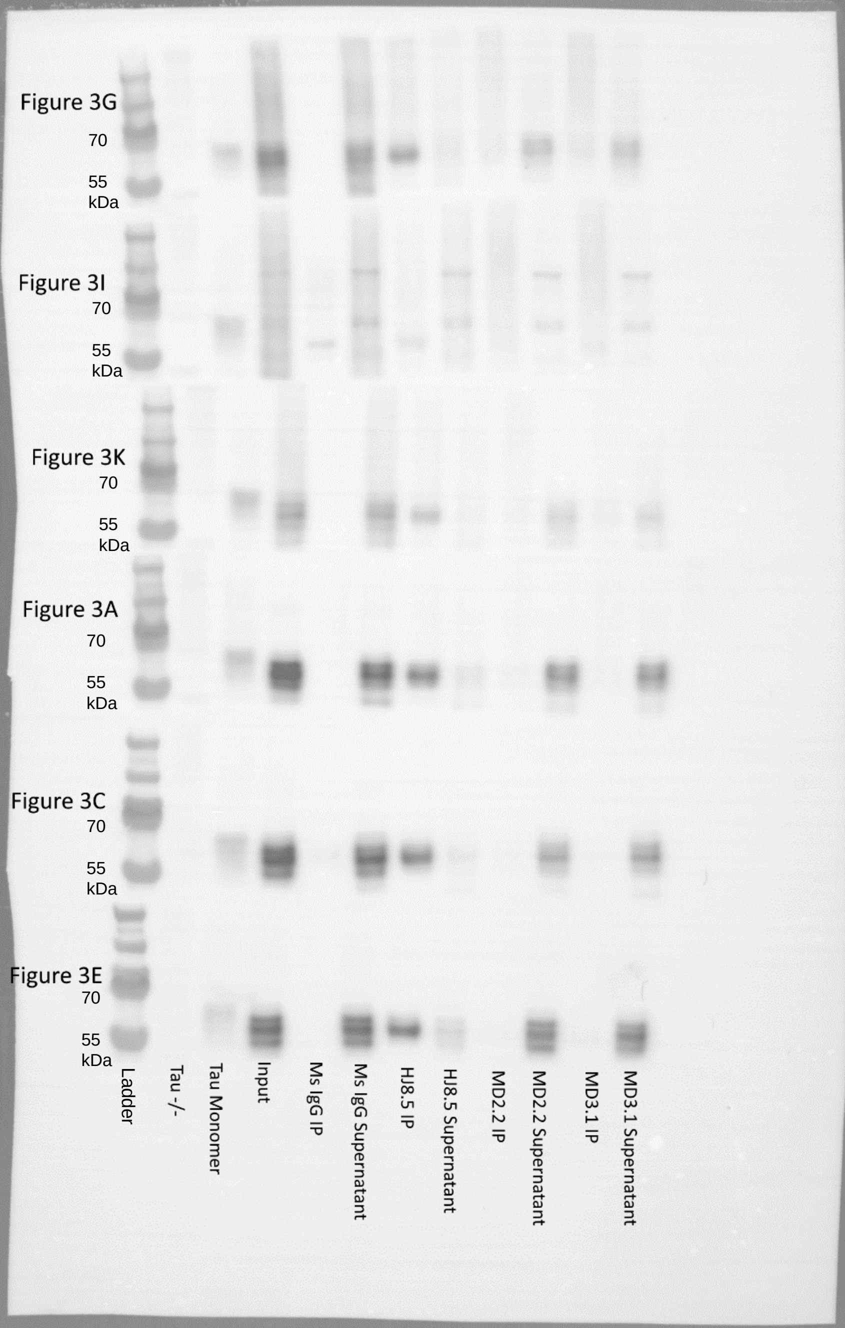
Figure 3G
70
55
kDa
Figure 3I
70
55
kDa
Figure 3K
70
55
kDa
Figure 3A
70
55
kDa
Figure 3C
70
55
kDa
Figure 3E
70
55
kDa
Ladder
Tau -/-
Tau Monomer
Input
Ms IgG IP
Ms IgG Supernatant
HJ8.5 IP
HJ8.5 Supernatant
MD2.2 IP
MD2.2 Supernatant
MD3.1 IP
MD3.1 Supernatant
